# Thirty-five years of the European Society of Hypertension: from past to future

**DOI:** 10.1097/HJH.0000000000003778

**Published:** 2024-06-26

**Authors:** Guido Grassi

**Affiliations:** Clinica Medica, Department of Medicine and Surgery, University of Milano-Bicocca, Milan, Italy

**Keywords:** educational activity, European Society of Hypertension, European Society of Hypertension excellence centers, European Society of Hypertension guidelines, European Society of Hypertension working groups, Hypertension

## Abstract

The European Society of Hypertension (ESH) was established 35 years ago. Since then, it promoted and coordinated a number of activities which include educational projects, epidemiological surveys and research investigations whose main focus includes different clinical and therapeutic aspects of the hypertensive disease. This article, which is based on data presented during the Presidential lecture held during the 33rd ESH Meeting in Berlin, will provide an overview of the main organization and structure of the ESH. Emphasis will be given to the relevance of the different society bodies, with a particular focus on the educational and research activities, such as the 15 working groups and the more than 120 excellence centers located in European and extra-European countries. Other main activities of the Society refer to the ESH Hypertension Specialist Program, the ESH Summer School, the ESH Young Fellow Program and the Annual Scientific Meeting of the Society. A special emphasis will be given to the central role of the Society in the organization of the various research projects and in the development and dissemination of the ESH Guideline document on hypertension diagnosis and treatment. Finally, the future perspectives of the ESH in the context of the European scientific framework will be highlighted.

## INTRODUCTION

The present article, which refers to the European Society of Hypertension (ESH) Presidential Lecture given during the 33rd ESH Meeting in Berlin, will be aimed at providing an updated overview of the main features of the ESH organization and structure, examining what it has been done during the past 35 years from the society constitution, what it is ongoing at the present time and what can be planned for future years.

**FIGURE 1:**
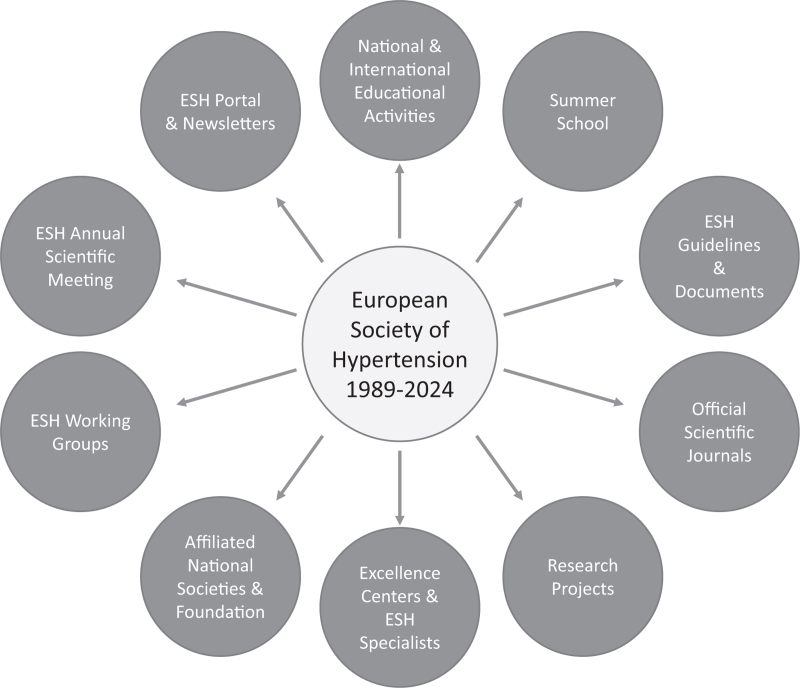
Scheme illustrating the main organization and activities of the European Society of Hypertension during the 35 years following its establishment in 1989.

Following an introductory paragraph dealing with a brief historical profile of the Society, the article will review the main framework of ESH with emphasis on the relative role of the different society bodies such as the scientific council, the board of the past presidents, the executive officers, the working groups as well as the excellences centers located in different European and extra-European countries (Fig. 1). This section of the manuscript will be followed by an in-depth analysis of the main educational activities currently ongoing under the leadership of the Society, including the main annual scientific meeting of the ESH. Special emphasis will be given to the central role of the Society in the various research projects and in the development and dissemination of the ESH guidelines document on hypertension diagnosis and treatment. This document, which has been recently published in 2023 [1], has received in its 2018 edition [2] a large number of citations, becoming one of the most quoted articles in the scientific literature worldwide. Finally, in the conclusive paragraph of the present article, a brief outlook to the future journey of ESH will be highlighted.

## HISTORICAL BACKGROUND OF EUROPEAN SOCIETY OF HYPERTENSION

At the beginning of 1980s, eminent clinicians and investigators in the field of hypertension realize the importance of planning meetings involving people from different European countries with the aim at improving scientific research, education and current clinical practice in hypertension and cardiovascular prevention. Following three successful ‘European Hypertension Meetings’, held in 1983, 1985 and 1987, in 1989, the ESH was established and it planned in the same year its first annual scientific meeting. In the following years, the ESH remarkably grew up, with organization of biennial (and from 1999 annual) scientific meetings, which have seen the participation of a large audience coming from European and extra-European countries, with a growing presence of representatives from American, Asian, Middle-East and North African countries. At the same time, the Society started to develop close relationships and expanding cooperations with national hypertension societies (so called ‘Affiliated Societies’), with the aim at establishing a long-lasting and well structured European network for scientific exchanges in hypertension, improving the quality of research as well as of the medical interventions in the disease prevention and clinical care.

In parallel, some hypertension societies and organizations from extra-European countries participated as ‘Associated Societies’ at developing programs and teaching activities promoted in conjunction with the ESH. Overall, at present 36 affiliated and 8 associated national societies work in close cooperation with the ESH. Furthermore, from its beginning, the ESH developed close relationships and collaborations with the International Society of Hypertension (ISH), sharing the official journal of the Society, the *Journal of Hypertension*, and having every 6-year period, a common annual scientific meeting with involvement of delegates of both societies. Finally, in very recent years, the ESH also became an important partner of the European Parliament, with specific reference to meeting with members of the European political institutions devoted to plan healthcare and global health strategies in the field of cardiovascular prevention.

## ORGANIZATIONAL FRAMEWORK OF EUROPEAN SOCIETY OF HYPERTENSION

The ESH Scientific Council represents the most important body of the Society, with the duty of supervising its main educational, research and organizational activities. It is structured as having 11 members, including officer bearers such as the president, the vice-president, the secretary, the treasurer and the officers at large, which are appointed with specific responsibilities in different areas of interest for the ESH. These include clinical specialist activities, excellence centers’ organization, coordination of the working groups, planning of the annual summer school and educational/research activities designed by the Society in conjunction with other national Societies, ISH and World Hypertension League (WHL). Many of these activities will be described more in detail in the following paragraphs.

Fifteen working groups, listed in Table 1, have been designed and organized to expand interest and investigation in specific areas of experimental and clinical hypertension, particularly when the research programs may require the expertise of different institutions and centers located in various European countries. The main goals of the Working Group's activity include the spread of excellence on hypertension and other related major cardiovascular diseases to reduce hypertension-induced morbidity and mortality across Europe, as well as the exchange of scientific information, promotion of research projects, and definition of appropriate recommendations for ESH and affiliated society programs.

**TABLE 1:** Working groups of the European Society of Hypertension

1. Blood Pressure Monitoring and Cardiovascular Variability
2. Endocrine Hypertension
3. Hypertension and the Heart
4. Diabetes and Metabolic Risk Factors
5. Hypertension and the Kidney
6. Large Arteries
7. Hypertension and the Brain
8. Small Arteries
9. Hypertension in Children and Adolescents
10. Lifestyle, Cardiovascular Pharmacotherapy and Adherence
11. Hypertension in Low Resource Settings
12. Device-based Treatment of Hypertension
13. Hypertension in Women
14. Environment in Hypertension
15. Hypertension in Older Adults

Although all the 15 working groups of ESH are very active in their respective areas of interest, a special mention should deserve the working group ‘Hypertension in Children and Adolescents’, which developed in the past years’ important editions of the Guidelines on Hypertension Diagnosis and Treatment in the Pediatric Age, the latest document being released in 2016 [3]. Other ESH working groups very active in their fields and worthy to be mentioned include the working groups on ‘Blood Pressure Monitoring and Cardiovascular Variability’ and ‘Small Arteries’. The former working group published in 2021 the Consensus Document on Practice Guidelines for Office and Out-Of-Office Blood Pressure Measurements [4] and vey recently the Consensus Statement on the Assessment and Management of Exaggerated Blood Pressure Response to Standing and Orthostatic Hypertension [5]. The working group on ‘Small Arteries’ provided last year a well structured document on microvascular inflammation in hypertension [6], and this year, a paper focused on the innovative therapeutic strategies which may exert favorable effects on the vascular inflammatory injury in the high blood pressure state [7].

In 2005, the ESH established an initiative for building a European network of hypertension excellence centers, which at present includes more than 120 institutions, located in 21 European and in 7 extra-European countries [8,9]. Main aim of this network is to provide a stable and organized platform of clinical institutions for providing the highest level of both inpatient and outpatient hypertension care, thereby contributing to facilitate hypertension prevention and control. Specific tasks for the ESH excellence centers include support for an optimal care of patients with hypertension, determination of the clinical and therapeutic standards for hypertension diagnosis and treatment, improvement of blood pressure control, planning of continuing medical education campaigns and implementation of experimental, epidemiological and clinical investigations and surveys with publication of the results in main scientific journals. Ongoing research projects supported by ESH include the ESH Atrial Fibrillation Research Project, the European Fibromuscular Dysplasia (FMD) Registry, the Blood Pressure Control Study (BP-CON-ESH), the ESH Registry of Hypertensive Urgencies and Emergencies (ESH-URGEM) and the Masked-uncontrolled Hypertension Management (MASTER) Study. In addition to the above-mentioned goals, global tasks for ESH excellence centers include the active cooperation with national societies and representative of general practitioners to define new hypertension control strategies and registries of specific hypertension-related diseases, renal denervation and other invasive therapeutic procedures.

## EDUCATIONAL ACTIVITIES OF EUROPEAN SOCIETY OF HYPERTENSION

Educational activities of ESH, which are supported by the Society through an important institution such as the ESH Foundation, include the ESH portal and e-learning programs, which have been successfully developed in 2009–2020 and underwent implementations in recent years. A further educational program is represented by ESH Scientific Newsletters, which were issued on a regular basis since 2000, being recently replaced by ESH Clinical Update and News with the aim at providing updated views on novel or controversial issues and/or specific topics with major clinical and therapeutic relevance.

Other major educational activities of the society include the ESH Hypertension Specialist Program, the Summer School and the Annual Scientific Meeting of the Society. As far as the Specialist Program, it should be emphasized that this educational project allows to train future specialists through teaching courses and close interactions with physicians working in the previously mentioned excellence centers in order to improve the diagnostic and therapeutic approach to specific clinical hypertensive phenotypes, such aa severe, resistant and secondary forms of hypertension [8]. The program, which specifically involves the general practitioner hypertension specialist and the hospital hypertension specialist, has seen about 1000 certifications since its beginning in 2000 [8].

The ESH Summer School represents the main annual educational event of the Society. The program, initiated in Spain in 1995, was followed every year by educational courses at which participated a large number of young doctors selected by the national societies and coming from European and extra-European countries. Every year, the program of the Summer School includes main lectures from well known investigators and clinicians working in European countries with the attempt to provide state-of-the-art reviews on major topics of clinical interest. The program also includes the active participation of the attendees, who are asked to present and discuss intriguing clinical cases. Specific editions of the Summer School have ben also organized in recent years in extra-European countries, such as in Latin America, in close cooperation with the American Society of Latin America (LASH), and in China, in cooperation with the Chinese Hypertension League (CHL).

The Annual Meetings of ESH represent important rendezvous of the Society during which lectures, debates and original communications are scheduled in the program. Guidelines on hypertension diagnosis and treatment have been also periodically presented at the annual ESH conference. A peculiar feature of the annual meetings, which has been implemented in the more recent conference editions, is represented by the ESH Young Fellows Program, finalized at increasing the active participation of young investigators and clinicians with the inclusion in the main program of the meeting of young fellow sessions. Postgraduate schools, active participation at the organization of annual conferences represent further institutional endeavors of the ESH Young Fellows group, whose core is represented by 16 young investigators from 15 European and extra-European countries.

## EUROPEAN SOCIETY OF HYPERTENSION GUIDELINES

Historically, essential hypertension represents one of the first clinical conditions for which diagnostic and therapeutic clinical guidelines have been developed. The first official guideline paper dates back to 1977 when the Joint National Committee in the United States released the document, which was revised few years later in a new paper jointly issued by an ‘ad hoc’ Committee of the WHO in conjunction with the International Society of Hypertension (ISH). During the last decade of the XX century, updated versions of the guidelines document have been prepared by the previously mentioned organizations in collaboration with American scientific societies. The first guideline document prepared by European Societies dates back to 2003, when the ESH together with the European Society of Cardiology jointly signed the European guidelines document [10]. New guidelines followed in 2007, 2013 and 2018 [2,11,12], the latter being as previously mentioned, the most frequently cited paper in the worldwide medical literature. The new guidelines document, released during the 32nd European Meeting on Hypertension held in Milan in 2023 and published in the *Journal of Hypertension*, represents the most updated document on the diagnosis and treatment of hypertension [1].

Although an in-depth analysis of the guidelines is beyond the scope of this article, it should be mentioned that the 2023 guidelines document addresses a number of critical issues in the field of hypertension diagnosis and treatment, also offering to the readers elements of novelties [1,13,14]. These include, for example, the thresholds and targets of the blood pressure-lowering intervention, the relevance of out-of-office blood pressure measurements, the modern quantification of cardiovascular risk, the greater use of organ damage measures, the upgrading of beta-blocking drugs to the level of major antihypertensive agents and the wider choice of drug combination between major drugs, with confirmation of their strategic role as first-line and subsequent treatment in the majority of patients. Finally, emphasis has been given to the treatment strategies based on renal denervation, particularly but not exclusively in drug-resistant hypertension, and to the patient's follow-up modalities, including the careful assessment of nonadherence as a fundamental barrier to the long-term blood pressure control.

Finally, given the importance of primary care providers in the diagnostic and therapeutic approach to the hypertensive state, ESH has decided to provide a novel concise format of the guidelines, the so called ‘2024 Clinical Practice Guidelines’, for implementation into current clinical practice [15]. The four pillars of these practice guidelines include the accurate diagnostic approach, the assessment of the patient's cardiovascular risk profile, the careful selection of the treatment strategy and the long-term evaluation of the blood pressure response [13].

## PERSPECTIVES AND CONCLUSION

In the future years, the Society will be asked to strengthen the collaboration with the national as well as with the other international scientific societies working in the area of hypertension. Of great importance will also be the growth of the young fellows of the Society which will represent the next core generation of ESH. Finally, two major additional future goals of the ESH activities will be pursued. First, to make the ESH as a major counterpart of the professional growth of each Society member. Second, on the other hand, to achieve established improvements in the prevention and treatment of the hypertensive disease in current clinical practice.

## ACKNOWLEDGEMENTS

G.G. would like to acknowledge the fundamental role of the Past Presidents, Council Members and Executive Officers of the ESH who succeeded one another during the 35 years of life of the ESH for their contribution to the development and progress of the Society in its various activities. A special thanks to the ESH Coordinator, Mrs Mandy Elgner for her continuous help during the years in monitoring as well as in improving the Society organization.

### Conflicts of interest

There are no conflicts of interest.
